# A Case of Unsuspected Peritoneal Mesothelioma Occurring with Colonic Adenocarcinoma Masquerading as Peritoneal Metastases

**DOI:** 10.1155/2014/838506

**Published:** 2014-05-20

**Authors:** Wei Xie, Linda K. Green, Rishi A. Patel, Syeling Lai

**Affiliations:** ^1^Department of Pathology and Immunology, Baylor College of Medicine, One Baylor Plaza, Houston, TX 77030, USA; ^2^Department of Pathology, Michael E. DeBakey VA Medical Center, 2002 Holcombe Boulevard, Houston, TX 77030, USA

## Abstract

We report a case of synchronous primary colonic adenocarcinoma and malignant mesothelioma. A 61-year-old male presented with a six-month history of fatigue and weight loss. An abdominal computed tomography (CT) scan showed a 5.8 cm partially obstructing mass in the cecum with ascites and peritoneal thickening. A biopsy of the large mass showed an adenocarcinoma. Because the patient was clinically thought to be a T4 colon carcinoma with peritoneal metastatic lesions (M1), prior to initiating chemotherapy, a debulking right hemicolectomy was performed. Resection of the colon and ileum revealed a T3N0 colonic mucinous adenocarcinoma and concurrent diffuse malignant peritoneal mesothelioma. Presenting synchronous colonic and peritoneal mesothelial primary malignancies are exceedingly rare but must be considered to prevent incorrect clinical staging.

## 1. Introduction

There are 102,480 new cases of colon cancer diagnosed every year in the United States. Approximately 50,830 patients die of colorectal cancer, accounting for 9% of all cancer death [1, 2]. Peritoneal malignant mesothelioma (MM) is an extremely rare and aggressive tumor. Its incidence rates ranging between 0.5 and 3 cases per 1,000,000 males and between 0.2 and 2 cases per 1,000,000 females in developed countries [3, 4]. Synchronous colonic adenocarcinoma and coexistent primary MM in patients are extremely uncommon. We report a case of colonic adenocarcinoma and unsuspected concurrent primary peritoneal MM, with a detailed clinical, pathologic, and immunohistochemical study of this unusual malignancy coexistence. It is critical to keep in mind that colonic adenocarcinoma can present with coexisting peritoneal MM since the staging, surgical interventions and additional treatment modalities can differ tremendously.

## 2. Case Report

**Figure 1:**
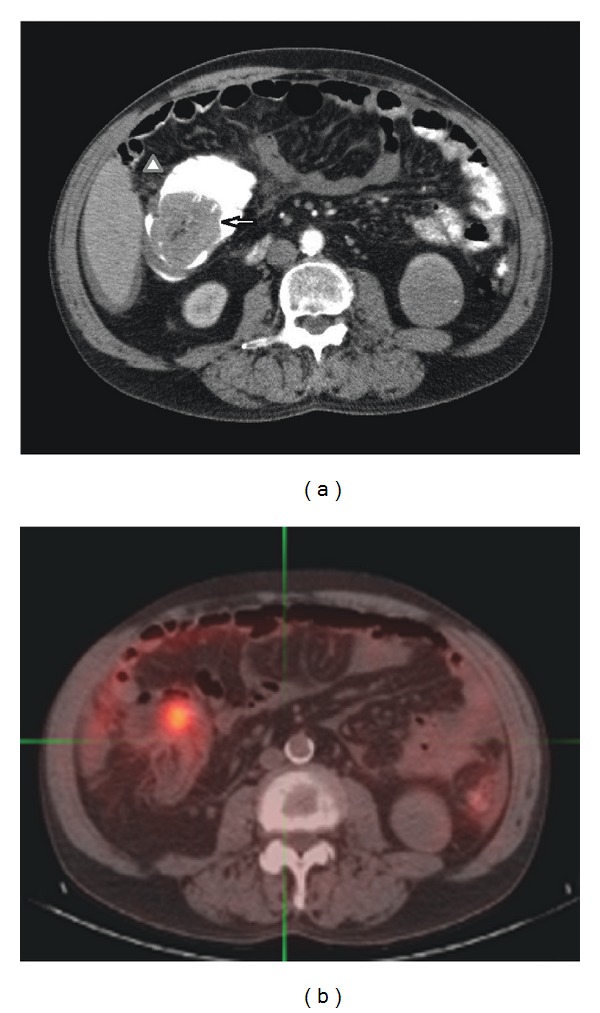
Abdominal imaging. (a) Computed tomography and (b) positron emission tomography scans showed a 5.8 cm partially obstructing cecal mass (arrow) with ascites and peritoneal thickening.

A 61-year-old man presented with a two-year history of worsening diarrhea, six months of fatigue, and weight loss. He had a 3-week history of abdominal pain. His past medical history was significant for coronary artery disease, hypertension, mitral valve repair, congestive heart failure, chronic obstructive pulmonary disease, and 40 pack year tobacco abuse. He was a Navy Veteran with an unknown service record. He was a retired quality control factory worker who at one time worked in construction. Although all factors could have had potential exposure to asbestos, he did not have a clear documented source of exposure to asbestos. He had a positive fecal occult blood test. Colonoscopy revealed a 5 cm circumferential mass in the ascending colon at the hepatic flexure consistent with a primary malignancy. A subsequent endoscopic biopsy of the mass revealed an adenocarcinoma arising in a tubulovillous adenoma. An abdominal computed tomography (CT) scan showed a 5.8 cm partially obstructing cecal mass (Figure 1(a)), ascites, and two left renal masses (2.1 cm and 4.5 cm). In addition, there were areas of associated fat stranding around the cecum and mesenteric fat stranding adjacent to the splenic flexure of the transverse colon. The peritoneal lining of the left abdomen showed focal irregularity. These findings were thought to represent peritoneal implants from the adenocarcinoma. Positron emission tomography (PET) scan also confirmed a large cecal mass and demonstrated focal fludeoxyglucose (FDG) uptake in the descending colon which was suspicious for metastatic disease (Figure 1(b)). There was no evidence seen on CT or PET scan of distant organ metastases. The radiographic and PET findings clinically staged him as a T4 unresectable adenocarcinoma of the colon with peritoneal metastasis (M1). Significant laboratory values included leukocytosis with a white blood cell count of 14.5 K/cmm (reference range: 3.5–10 K/cmm), an elevated serum CEA of 12.7 ng/mL (reference range: 0–10 ng/mL), and a low blood urea nitrogen (BUN) of 3 mg/dL (reference range: 7–19 mg/dL).

Since he developed obstructive symptoms two months after his initial presentation and at the recommendation of his treating Oncologist, a palliative hand-assisted laparoscopic right hemicolectomy was performed in order to decrease the tumor burden to optimize the effects of his planned chemotherapy treatments. During the procedure, diffuse peritoneal implants thought to be consistent with suspected carcinomatosis were identified by the surgeon and approximately 800 mL of thick peritoneal fluid was evacuated. The patient tolerated the procedure without any overt complications. After his final diagnosis, he was offered chemotherapy for his colonic adenocarcinoma and unsuspected mesothelioma, but, with his unsure prognosis related to his mesothelioma, he declined further treatments. He chose to transfer his care to another facility. In follow-up, he continues to suffer from massive ascites with periodic therapeutic paracentesis but is alive at 56 months since his diagnosis.

### 2.1. Pathology

#### 2.1.1. Gross Findings

The right hemicolectomy specimen consisted of a segment of right colon and terminal ileum with attached appendix and separate portion of omentum. In the colonic mucosa, 7.2 cm from ileocecal valve and 13.4 cm from distal margin, there was a tan-red to pink polypoid, nearly circumferential mass, which measured 6.5 × 5.3 × 4.9 cm. On cross section, the mass was tan-white and friable with a mucinous appearance. The mass extended through the underlying muscularis propria to the serosa and surrounding mesenteric adipose tissues. Overlying the external surface of the mass, there was a tan-red area of serosal puckering which measured 1.5 × 1.5 cm. The remainder of the serosa of ileum (including the proximal margin), appendix and colon serosa, and surrounding mesenteric adipose tissues was diffusely red, gritty, and scabrous with small nodules ranging from 0.2 cm to 0.6 cm. These multiple lesions were presumed to represent serosal metastases from the adenocarcinoma. Within the attached mesenteric fat, there were multiple tan-pink, rubbery lymph nodes ranging from 0.2 cm to 0.7 cm. The omentum was very abnormal and showed a “bumpy,” tan to yellow nodular appearance.

#### 2.1.2. Histologic Findings

**Figure 2:**
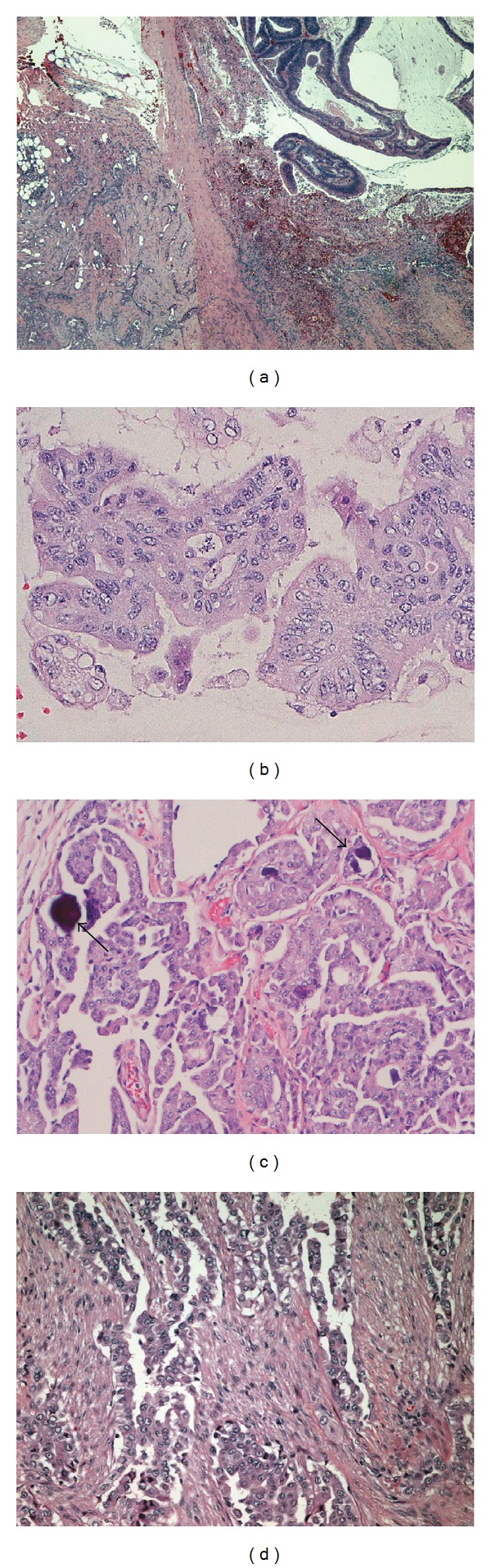
Histopathology of tumors. (a) Malignant mesothelioma (left) and adenocarcinoma in mucin pool (right). (b) Adenocarcinoma with atypical glands. (c) Mesothelioma showing psammoma bodies (arrows) and papilla with fibrovascular cores. (d) Mesothelioma demonstrating tubuloglandular structures.

Microscopic sections of the colonic mass showed a full thickness infiltrating adenocarcinoma comprised predominantly of irregularly infiltrating fused glands with focal tubular structures and small individual cell clusters. There was a mucinous adenocarcinoma component with large lakes of extracellular mucin and suspended tumor glands. This pattern comprised more than 50% of the tumor (Figures 2(a)-2(b)). In areas of the tumor, there was an intense desmoplastic response to the tumor cells with surrounding “plump” spindle cells. Focal necrosis was present. The tumor cells had large, elongated “cigar-shaped” nuclei with irregular nuclear rims and hyperchromatic chromatin. There was brisk mitotic activity. The tumor invaded through the muscularis propria and focally infiltrated the serosal fibrous tissue and adjacent adipose tissues. The mass was arising from an associated tubulovillous adenoma. In the thirty-one lymph nodes examined, no metastatic adenocarcinoma was found. The National Comprehensive Cancer Network (NCCN) stage of this invasive mucinous adenocarcinoma was T3, N0.

Examination of the grossly visible serosal gritty areas with nodules showed a completely different pattern from the colonic mucinous adenocarcinoma. The cells resembled mesothelial cells. The tumor was composed predominantly of infiltrating and superficial collections of cells that formed papillary structures with identifiable fibrovascular cores and psammoma bodies (Figures 2(a) and 2(c)). There were cells forming tubules and many single, infiltrating cells (Figures 2(a) and 2(d)). The tumor cells had hyperchromatic, round to polygonal nuclei with irregular nuclear rims and prominent nucleoli. They had abundant eosinophilic cytoplasm (Figures 2(c)-2(d)). The tumor cells infiltrated into the intestinal muscularis propria and extensively infiltrated the omentum and mesenteric fat.

#### 2.1.3. Immunohistochemistry

**Figure 3:**
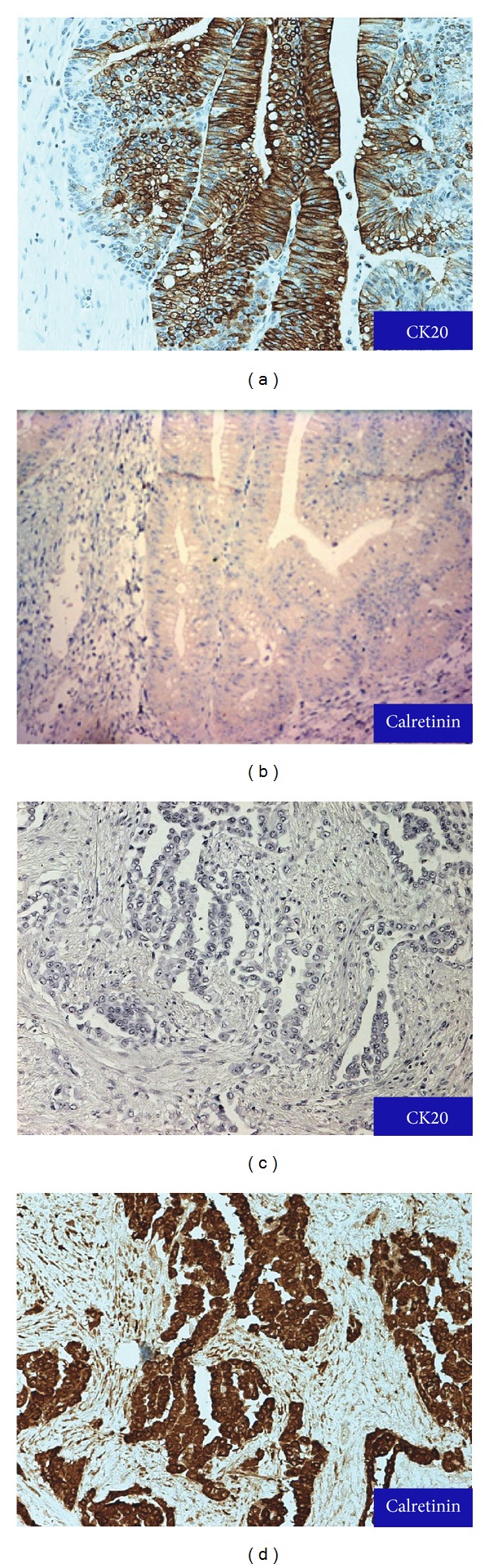
Immunohistochemical staining. (a, b) Adenocarcinoma stained with CK20, but not calretinin. (c, d) Malignant mesothelioma showed negative staining with CK20 and positive immunoreactivity with calretinin.

Immunohistochemical stains were performed to compare the two neoplasms using automatic tissue staining: Bond Polymer Refine Detection System (Leica Biosystems, Newcastle, UK) with multiple antibodies according to manufacturer's instruction (Vantana Tucson, Arizona, USA). The colonic adenocarcinoma was positive for CK20, monoclonal CEA, and villin (Figure 3(a)), but negative for calretinin, CK5/6, and CK7 immunostaining (Figure 3(b)). In contrast, the separate mesothelial-like masses in the serosa showed no staining with CK20, monoclonal CEA, and villin (Figure 3(c)). Unlike the adenocarcinoma, there was positive staining with calretinin, CK5/6, and CK7 (Figure 3(d)). Both tumors showed no staining with TTF-1, PAX-2, CD10, RCC, P504S, and vimentin. Based on the morphology and immunostaining profile, the second tumor was diagnosed as multifocal peritoneal malignant mesothelioma, epithelioid type.

## 3. Discussion

Colorectal cancer is the second leading cause of cancer-related deaths in the United States and the third most common cancer in men and women. In contrast, mesothelioma, a neoplasm arising from the mesothelial lining cells of pleura, peritoneum, pericardium, and tunica vaginalis are uncommon and peritoneal MM is an extremely rare tumor [5]. It was first described in 1908 by Miller and Wynn [6]. Most malignant mesotheliomas arise from the pleura in relation to occupational exposure to asbestos. It was exceeding rare before 1930, when the industrial use of asbestos expanded. With the delay in development from exposure to asbestos, the incidence has increased in the past 2 decades. Only 20–33% of all mesotheliomas arise from the peritoneum itself. Each year, only approximately 250 to 500 new cases of peritoneal mesothelioma were diagnosed in the United States [3, 7, 8]. It can occur at any age but is more common in 50–69-year-old men due to occupational exposure of crocidolite variety of asbestos. Interestingly, 50% of patients with peritoneal MM have no documented asbestos expose. Other reported risk factors include prior radiation ports, exposure to irritants (thorium, talc, erionite, or mica), familial Mediterranean fever, diffuse lymphocytic lymphoma, and simian virus [3, 9]. Although overall mesothelioma is more common in men, higher proportions of women develop peritoneal mesothelioma [3, 10]. Common postulated associations with the development of colonic adenocarcinoma include transformation from adenomatous polyps, familiar adenomatous polyposis, dietary factors, environmental factors, smoking, family history of colon cancer, and inflammatory bowel disease. To the best of our knowledge, this is the first case report of synchronous colonic adenocarcinoma and coexistent primary peritoneal MM in a male patient. Because it is so rare, it is difficult to postulate why these tumors occurred together (by chance, environmental factors or shared underlying genetic abnormalities). Rare cases of peritoneal mesothelioma after radiation therapy for testicular seminoma, ovarian teratocarcinoma, and cervical cancer have been reported previously [11–15]. Those mesotheliomas were considered secondary to radiation therapy induction [11–15]. The coexisting colonic adenocarcinoma and MM in the current case fulfill the diagnostic criteria of multiple primary malignant neoplasms, which is a rare entity [5, 16]. The two tumors showed distinctive morphology and arose from different tissue origin as evidenced by histologic examination and immunohistochemical studies [10].

Patients with peritoneal MM can present with abdominal distention, fatigue, weight loss, and organ impairment such as bowel obstruction as seen in our patient [17]. These manifestations are common abdominal neoplasm syndromes which are indistinguishable from those caused by colon cancer. The most common presenting symptom in peritoneal MM is abdominal pain as seen in our patient. This is an unusual presenting symptom in colonic adenocarcinoma. Peritoneal MM patients can also present with paraneoplastic syndromes such as thrombocytosis, hypoglycemia, venous thrombosis, paraneoplastic hepatopathy, and a wasting syndrome. In this case, CT and PET scans showed partially obstructing cecal mass with ascites, mesenteric fat stranding, and peritoneal irregularity. Although these findings can be seen in mesothelioma, they are not typical and nonspecific. The possibility of mesothelioma as differential diagnosis could be more likely raised if patients had a history of asbestos exposure and diffuse peritoneal encasing/thickening without other solid organ primary tumors. It is difficult to make a diagnosis of MM with only nonspecific symptoms and overlapping imaging. With previous biopsy proven colonic adenocarcinoma and lack of history of asbestos exposure in our patient, the radiographic features were considered as metastases from the primary colonic cancer. Therefore, this case was clinically staged as T4 for colonic serosal involvement of adenocarcinoma and peritoneal metastasis (M1). As discussed in other previous reports, the role of PET or CT scans in peritoneal mesothelioma diagnosis is unclear [18]. The initial therapy plan for this case was palliative chemotherapy for metastatic adenocarcinoma. Surgical intervention was performed later only for palliative release of patient's obstructing symptoms. MM is an incidental pathological finding. If palliative procedure was not performed, detection of MM could have been entirely missed in this case.

The lesson we learned from this case is to keep in mind that MM can coexist with adenocarcinoma and tissue diagnosis is crucial for accurate staging colon cancer before further management. Cytologic examination of ascitic fluid could be an initial assessment to differentiate between peritoneal metastatic adenocarcinoma and mesothelial proliferative lesion. If findings were negative or inconclusive, subsequent CT or ultrasound-guided biopsy or fine needle aspiration of mesentery, peritoneal, and omental irregular area/nodules may as well be used to rule out metastatic carcinoma and assess for mesothelioma. In rare situation of suspicious lesion, laparoscopic exploration with tissue biopsy should lead to definitive diagnosis.

Pathologic diagnosis of peritoneal MM is based on gross findings, microscopic patterns, and immunostaining profile. Grossly, MM usually presents as multiple nodules, plaques, or diffuse thickening of the peritoneum. Solitary mass is uncommon, which is usually benign. MM exhibits variable microscopic types, predominantly epithelioid, sarcomatoid, and mixed biphasic types. The most common type is epithelioid, which shows tubulopapillary, glandular, adenomatoid, microglandular, and solid patterns. Tubulopapillary and glandular patterns as seen in our case consist of a mixture of papillary structures lined with atypical flat, cuboidal, or polygonal cells with fibrovascular cores, glands, and tubules. The tumor papilla needs to be differentiated from primary peritoneal cavity carcinoma extension or metastatic carcinoma with papillary pattern, such as papillary urothelial carcinoma, papillary renal cell carcinoma, and pulmonary adenocarcinoma. In female patients, ovarian papillary serous carcinoma (PSC) is of concern. Differential diagnosis for neoplastic glands and tubules also includes gastrointestinal, pancreatobiliary, and pulmonary adenocarcinomas. Our case had biopsy proven colonic adenocarcinoma. It was easy to assume that the peritoneal findings were due to colonic carcinoma spread through vessels or lymphatic spaces (carcinomatosis). Careful microscopic examination, awareness of different patterns, extensive sampling, and further ancillary studies are critical to identify secondary malignancies arising from an entirely different origin. Exuberant mesothelial hyperplasia, which is more frequently encountered in the peritoneum rather than the pleura, may mimic MM. Our case showed peritoneal tumor with serosa stromal fibroadipose tissue and muscularis propria invasion, which is a diagnostic feature of malignancy in MM. Other features distinguishing MM from hyperplasia include (1) dense cellularity, (2) complex papillae, (3) tubules, (4) disorganized growth, (5) expansion of nodules, (6) cytologic atypia, and (7) necrosis not seen with hyperplasia. Of note, although demonstration of invasion is the key feature of a malignant diagnosis and solitary mesothelial proliferation is usually considered benign, there are exceptions in both entities. In rare situation, the presence of invasion is not required for the diagnosis of MM in solid fragments of mesothelial tumor with histologic features of malignancy [1].

There is an important subtype of mesothelioma which may be difficult to identify from merely reactive mesothelial proliferation. A well-differentiated papillary mesothelioma of the peritoneum is generally noninvasive, lined with uniform bland mesothelial cells. It does not have multilayering and there is an absence of the common cytologic features associated with malignancies. It is more common in young women. Although it may recur or progress to a more aggressive clearly malignant mesothelioma, this tumor usually has an excellent prognosis and treatment is less aggressive than that applied to MM [1, 18]. It may therefore be overlooked in cases in which primary resection of colon and peritoneum is for a separate malignant or benign tumor.

Highly selective panels of immunohistochemical (IHC) stains are needed to distinguish mesothelioma from adenocarcinomas of the gastrointestinal tract. Because both tumors may fail to stain uniformly with commonly known markers, several specific mesothelioma stains and several common adenocarcinoma stains should be used. Often each laboratory will devise their unique staining panels. None of the antibodies used are 100% specific or 100% sensitive. Approximately 10% false positive reactions can occur which may be related to fixation or techniques. Common mesothelial IHC panel includes calretinin, WT-1, CK7, CK5/6, and vimentin. D2-40 also stains with mesothelioma. Colonic adenocarcinoma can stain with CK20, villin, monoclonal CEA, CDX-2, and Moc31. We used mesothelial IHCs CK5/6, calretinin, and adenocarcinoma IHCs monoclonal CEA. It should be noted that monoclonal CEA and WT-1 may stain ovarian PSC. There is infrequent staining CEA in PSC and positive reaction of WT-1 in both mesothelioma and PSC. Our patient was male, so PSC was not in the differential diagnosis. Our case showed typical MM and adenocarcinoma staining patterns with our IHC stains. We also included additional carcinoma IHC stains to rule out that the peritoneal tumors were of different origins: CK7, CK20, and villin for gastrointestinal, urothelial primary tumor, TTF-1 for lung adenocarcinoma, CD10, RCC, and vimentin for clear cell renal cell carcinoma, and P504S and PAX-2 for papillary renal cell carcinoma.

Asbestos is a well-known carcinogen associated with MM [3, 7, 8]. Mesothelial cells readily proliferate in response to pleural or peritoneal injury and stimulation of growth factors [9]. In this case, patient has no history of asbestos exposure. According to the guideline for pathologic diagnosis of MM, history of asbestos exposure is not required for making the diagnosis of MM [18]. No common risk factors of colon cancer other than history of smoking were evident in this patient. It remains unknown if colonic adenocarcinoma stimulates mesothelial proliferation or if these two tumors coexist and grow under different signaling pathway. Etiology of cancer is multifactorial. Changes in tumor suppressor gene and/or oncogene could have field effect involving multiple organs. Apostolou et al. discovered absence of posttranscriptional RNA modifications of B-cell lymphoma/leukemia 10 (BCL10) in human malignant mesothelioma and colorectal cancer [19]. Adenomatous polyposis coli (APC) gene has mutation in approximately 80% colon adenocarcinoma. In the study, by Abutaily et al., the APC gene expression was also found to be altered in some malignant mesothelioma [20]. Genetic alteration may have played an important role in tumorigenesis of both tumors in our case.

Complete resection with excision of all visible peritoneal tumors followed by subsequent chemotherapy is a commonly used treatment for limited stage MM [17, 18]. Survival is poor with median survival of 10 months and 5-year survival of 16% [3]. A longer survival with a median longer than 50 months has been reported [3]. Our patient seems to have better survival (at least 62 months), but he had symptomatic treatment with periodic paracentesis to relieve his ascites followed by fluid replacement. There is no reliable tumor serum marker for the diagnosis and follow-up for recurrence. CT imaging modalities can be used for follow-up.

In conclusion, a review of the existing literature has shown that presenting synchronous colonic adenocarcinoma and coexistent primary peritoneal MM are exceedingly rare but must be considered to prevent incorrect clinical staging. Tissue examination to identify different morphologic patterns and confirm differentiation of tumor origin with immunohistochemistry is critical for definitive diagnosis.

## References

[1] Jemal A, Simard EP, Dorell C (2013). Annual Report to the Nation on the Status of Cancer, 1975–2009, featuring the burden and trends in human papillomavirus(HPV)-associated cancers and HPV vaccination coverage levels. *Journal of the National Cancer Institute*.

[2] Siegel R, Naishadham D, Jemal A (2013). Cancer statistics, 2013. *CA: A Cancer Journal for Clinicians*.

[3] Boffetta P (2007). Epidemiology of peritoneal mesothelioma: a review. *Annals of Oncology*.

[4] Taskin S, Gumus Y, Kiremitci S, Kahraman K, Sertcelik A, Ortac F (2012). Malignant peritoneal mesothelioma presented as peritoneal adenocarcinoma or primary ovarian cancer: case series and review of the clinical and immunohistochemical features. *International Journal of Clinical and Experimental Pathology*.

[5] Hassan R, Alexander R (2005). Nonpleural mesotheliomas: mesothelioma of the peritoneum, tunica vaginalis, and pericardium. *Hematology/Oncology Clinics of North America*.

[6] Miller J, Wynn WH (1908). A malignant tumour arising from the endothelium of the peritoneum, and producing a mucoid ascitic fluid. *The Journal of Pathology and Bacteriology*.

[7] Teta MJ, Mink PJ, Lau E, Sceurman BK, Foster ED (2008). US mesothelioma patterns 1973–2002: indicators of change and insights into background rates. *European Journal of Cancer Prevention*.

[8] Rodríguez D, Cheung MC, Housri N, Koniaris LG (2009). Malignant abdominal mesothelioma: defining the role of surgery. *Journal of Surgical Oncology*.

[9] Pelin K, Hirvonen A, Linnainmaa K (1994). Expression of cell adhesion molecules and connexins in gap junctional intercellular communication deficient human mesothelioma tumour cell lines and communication competent primary mesothelial cells. *Carcinogenesis*.

[10] Eisenstaedt JS (1938). Multiple primary malignant tumors. *Journal of the American Medical Association*.

[11] Tassile D, Roth AD, Kurt AM, Rohner A, Morel P (1998). Colon cancers and peritoneal mesothelioma occurring 29 years after abdominal radiation for testicular seminoma. A case report and review of the literature. *Oncology*.

[12] Antman KH, Carson JM, Li FP (1983). Malignant mesothelioma following radiation exposure. *Journal of Clinical Oncology*.

[13] Amin AMH, Mason C, Rowe P (2001). Diffuse malignant mesothelioma of the peritoneum following abdominal radiotherapy. *European Journal of Surgical Oncology*.

[14] Gilks B, Hegedus C, Freeman H, Fratkin L, Churg A (1988). Malignant peritoneal mesothelioma after remote abdominal radiation. *Cancer*.

[15] Babcock TL, Powell DH, Bothwell RS (1976). Radiation induced peritoneal mesothelioma. *Journal of Surgical Oncology*.

[16] Warren S, Gates O (1932). Multiple primary malignant tumors: a survey of the literature and statistical study. *American Journal of Cancer*.

[17] Sugarbaker PH, Welch LS, Mohamed F, Glehen O (2003). A review of peritoneal mesothelioma at the Washington Cancer Institute. *Surgical Oncology Clinics of North America*.

[18] Husain AN, Colby TV, Ordóñez NG (2009). Guidelines for pathologic diagnosis of malignant mesothelioma: a consensus statement from the International Mesothelioma Interest Group. *Archives of Pathology and Laboratory Medicine*.

[19] Apostolou S, Murthy SS, Kolachana P, Jhanwar SC, Testa JR (2001). Absence of post-transcriptional RNA modifications of BCL10 in human malignant mesothelioma and colorectal cancer. *Genes Chromosomes Cancer*.

[20] Abutaily AS, Collins JE, Roche WR (2003). Cadherins, catenins and APC in pleural malignant mesothelioma. *Journal of Pathology*.

